# Comparison of Clinical Effects and Physical Examination of Transforaminal and Caudal Steroid Injection With Targeted Catheter in Lumbar Radiculopathy: A Single‐Blind Randomized Clinical Trial

**DOI:** 10.1002/brb3.70067

**Published:** 2024-10-04

**Authors:** Farnad Imani, Faezeh Mohammad‐Esmaeel, Seyedeh‐Fatemeh Morsalli, Ali Ahani‐Azari, Mahzad Alimian, Nasim Nikoubakht, Azadeh Emami

**Affiliations:** ^1^ Pain Research Center, Department of Anesthesiology and Pain Medicine, School of Medicine Iran University of Medical Sciences Tehran Iran

**Keywords:** caudal, epidural, lumbar radiculopathy, steroid, targeted catheter, transforaminal

## Abstract

**Background:**

Transforaminal and caudal epidural injections are two methods of steroid injection in lumbar radiculopathy. Using a targeted catheter with the possibility of accessing the involved spinal roots and steroid administration selectively next to them during the caudal procedure may achieve the benefits of both transforaminal and caudal procedures. The aim of this study was to investigate the clinical effects and physical examinations of transforaminal steroid injection compared to caudal through a targeted catheter in lumbar radiculopathy.

**Methods:**

Fifty patients with lumbar radiculopathy candidates for epidural steroid injection were divided into transforaminal (T) and caudal (C) groups. Steroid injection under fluoroscopic guidance was performed in group T with the transforaminal method and in group C with the caudal method using a targeted catheter for each involved spinal nerve root. Pain intensity visual analog scale (VAS), Oswestry Disability Index (ODI), daily analgesic consumption, and physical examinations on four follow‐ups (before injection, second week, first and third month) were evaluated.

**Results:**

Pain score (VAS) and functional disability index (ODI) were similar in both groups, and there was no significant difference between the two groups (*p* > 0.05). The positive Lasègue test was significantly higher in the caudal group than in the transforaminal group only in the third month (*p* < 0.05). Other physical examinations in both groups did not have significant differences in all the follow‐ups. Moreover, there was no difference in the amount of analgesic consumption in the two groups. No complications were observed in both groups.

**Conclusion:**

This study showed that transforaminal and caudal steroid injection (with a targeted catheter) in patients with lumbar radiculopathy had similar effects in controlling pain and improving functional disability of patients in the short term. Cases of recurrence of positive Lasègue test in physical examinations in the long term (third month) in the caudal group may indicate the preference of the transforaminal approach.

**Trial Registration:**

Iranian Registry of Clinical Trials (IRCT) number: IRCT20111102007984N31

## Graphical abstract

• Transforaminal and caudal steroid injection (with a targeted catheter) in lumbar radiculopathy had similar effects in controlling low back and radicular pain and improving functional disability in the short term. • The cases of positive Lasègue test recurrence in the long term in the caudal group may indicate the preference for the transforaminal approach.

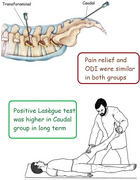

## 1 Introduction

The most common cause of lumbar radiculopathy is due to mechanical compression of spinal nerve roots, such as lumbar disc herniation (LDH) (Malik et al. 2022). Pain improves in many patients within a few weeks, and conservative, nonsurgical methods are always the first step of treatment (Lee et al. 2019). In cases resistant to conservative treatments, minimally invasive or surgical procedures can be performed. It has been shown in many studies that biochemical factors alone or together with mechanical pressures are important factors in the occurrence of pain. Epidural corticosteroid injection is one of the common percutaneous methods for treating these patients, which, by inhibiting the synthesis or release of some pro‐inflammatory mediators as well as creating reversible local anesthetic effects, can reduce inflammation and subsequently reduce pain (McLain, Kapural, and Mekhail 2005; Rivera 2018). Corticosteroid injection is performed by three common methods: interlaminar, transforaminal, and caudal. Although only interlaminar and caudal approaches can be performed blindly, performing these three methods with fluoroscopic guidance can increase the success rate and reduce the incidence of complications, such as intravascular injection (Husseini et al. 2020). Although the steroid injection in the transforaminal method is selectively next to the spinal nerve root, in the case of involvement of more than one root, it needs separate skin entry points to access each one (Bhatia et al. 2016). In this situation, although transforaminal injection using a consecutive needle may reduce the cost of consumables, the increase in the duration of the procedure and the time of fluoroscopy may negate this advantage. Steroid injection through the caudal, like the interlaminar method, is nonselective in the epidural space, but it has less risks such as dural tear. On the other hand, it may be less effective for higher lumbar levels. Therefore, using a targeted catheter in the caudal approach used for epidural neuroplasty with the possibility of accessing the involved spinal roots and selectively injecting steroids next to them may achieve the benefits of both transforaminal and caudal methods (Oh et al. 2014; Taheri et al. 2016). In addition, performing physical examinations to assess treatment outcomes may be helpful for other common measures such as pain scores (van der Windt et al. 2010). The aim of this study is to investigate the clinical effects (visual pain score, Oswestry Disability Index [ODI], and physical examinations) of caudal steroid injection through a targeted catheter compared to transforaminal in the management of lumbar radiculopathy.

## 2 Methods

**FIGURE 1:**
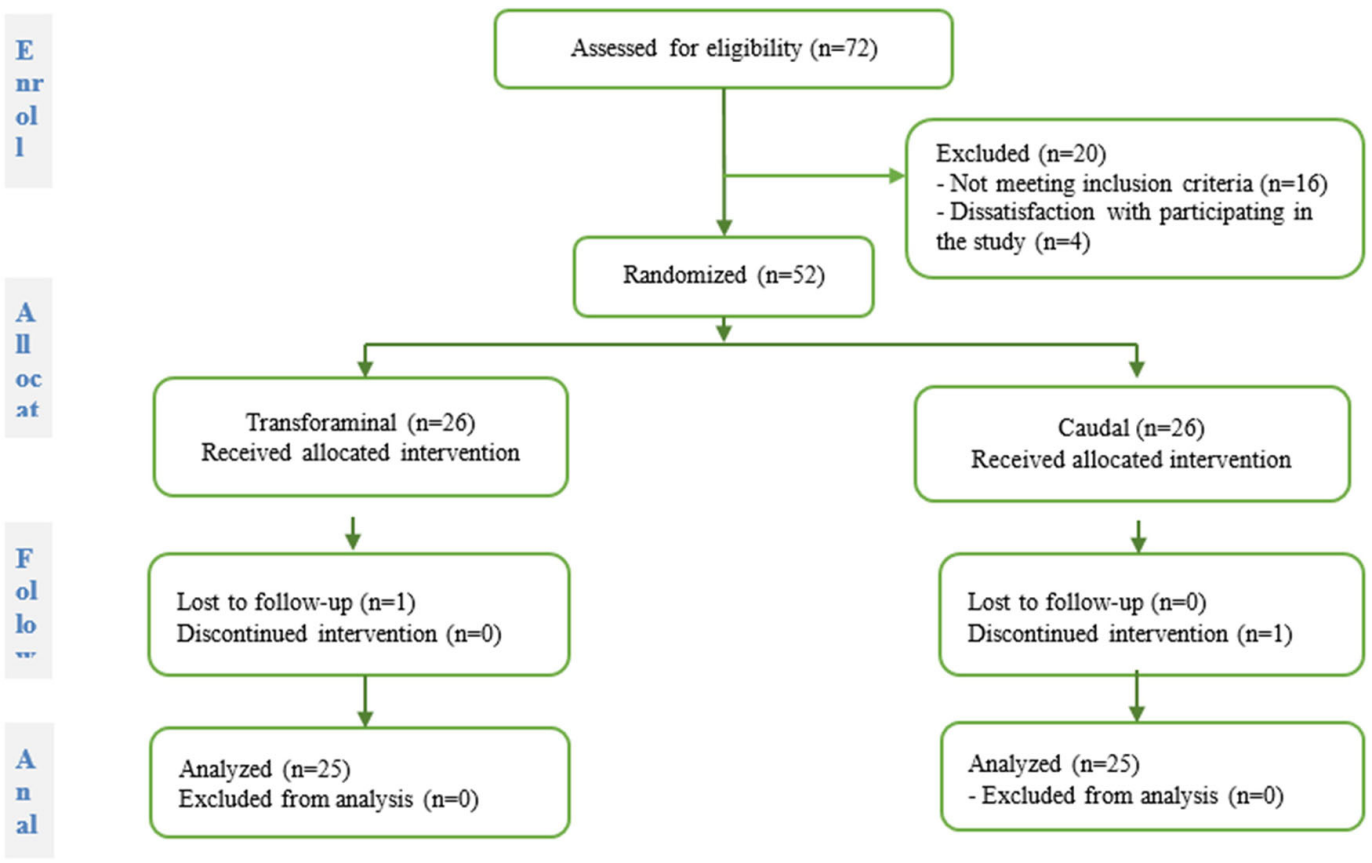
Flowchart of the present clinical trial.

In this single‐blind randomized clinical trial, patients referred to the pain clinic with lumbar radiculopathy due to LDH from February to September 2022 were studied according to the Consolidated Standards of Reporting Trials (CONSORT) guidelines (Figure 1). To calculate the sample size, the average comparison formula for two independent populations was used, and the maximum sample size was estimated to be 25 patients for each group, considering the confidence level of 95% and the power of 80% based on the average visual analog scale (VAS) score in previous studies:

$$n=\frac{{\left(z_{1}-\frac{\alpha }{2}+z_{1}-\beta \right)}^{2}\left(\sigma_{1}^{2}+\sigma_{2}^{2}\right)}{{\left(\mu_{1}-\mu_{2}\right)}^{2}}$$

Inclusion criteria include age 40–70 years, ASA class I or II, unilateral or bilateral chronic radicular back pain, moderate‐to‐high pain score (VAS ≥ 3), LDH at one to two lumbar levels based on MRI findings, and also physical examination and failure to respond to conservative treatments (including prescription of oral pain medications such as daily pregabalin, acetaminophen, diclofenac, and also physical therapy) for at least 3 months. Exclusion criteria include spinal deformity, tumor, fracture, spondylolisthesis more than grade II, severe stenosis (more than two‐thirds in axial cut), disc sequestration, local or systemic infection, coagulation disorders, drug abuse, history of allergic reaction to study drugs, history of severe mental illness, severe disorders or sudden onset of neurological complications such as urinary and fecal incontinence, history of lumbar spinal surgery, history of any steroids intake (e.g., oral, parenteral, transforaminal, and caudal routes), and patient refusal. After obtaining written informed consent, the patients were divided into two transforaminal (T) and caudal (C) groups by simple randomization method by a colleague who was not involved in the procedure. After inserting an intravenous catheter (20 G) for crystalloid injection, midazolam (1 mg) was injected for sedation. Monitoring included noninvasive arterial blood pressure measurement, electrocardiogram, and pulse oximetry. The intervention in both groups was performed in the prone position under local anesthesia with fluoroscopic guidance. In the transforaminal group (T group) in the oblique fluoroscopy view on the same side (15°–20°) after observing the Scottie dog, first the introducer needle is inserted under the intersection of the transverse process and the pedicle with the tunnel view, and through it, the blunt‐curved tip needle G20, 8.9 cm (Epimed, Dallas, TX, USA) is placed. The correct location of the needle tip was confirmed with the lateral fluoroscopy view (in the posterior superior quadrant of the foramen). A contrast medium (Visipaque 270) of 2 mL is injected, and if it spreads properly and there is no vascular spread, the injection solution for each level includes triamcinolone 20 mg (triamcinolone acetonide, Elixir, Iran), and 5 mL of ropivacaine 0.2% (Ropivacaine, Molteni, Giussano, Italy) was injected slowly. In the caudal group (group C), the sacral hiatus is determined in the lateral fluoroscopy view, and the RX‐2 Coudé epidural needle 16G, 10.6 cm (Epimed), enters the caudal epidural space through it. Then, the targeted Brevi‐XL radio‐opaque epidural catheter 19G, 35 cm (Epimed), is inserted and guided under the pedicle of the involved vertebrae. Injection of the same solution as the transforaminal group (triamcinolone 20 mg and 5 mL of ropivacaine 0.2%) was performed for each level. After the end of the intervention, the patients were transferred to recovery, and in the case of hemodynamic stability, the absence of sensory and movement disorders and consciousness, as well as fluid tolerance, they were discharged from recovery. The same conservative medications (pregabalin and analgesics) were continued for postoperative period as follows: Pregabalin 75 mg to all patients, and if the pain score (VAS) was more than 3, at first, oral daily acetaminophen 500 mg up to three times. If the VAS did not decrease to less than 3 after 48 h, oral diclofenac 50 mg daily up to two times was added to acetaminophen. The person responsible for follow‐up and data gathering and the patients were blinded to the randomization method and the intervention method. Visual analog pain score (VAS), ODI, daily analgesic consumption (acetaminophen and diclofenac), and physical examinations (Lasègue, Bonnet, Faber, Bragard, Psoas, tap, stoop, heel walking, and tip toe tests) on four follow‐ups (before injection, second week, first and third month) were evaluated. In the case of improvement of less than 50% in pain score and ODI, a second injection was performed with the same method as before, but in the case of no response or worsening of symptoms, the patient was referred to a neurosurgeon.

### 2.1 Statistical Analysis

The data were analyzed using SPSS version 22 statistical software. Central indices (mean and standard deviation) were used to report quantitative variables. Frequency (%) was used to report qualitative variables. The normality of the distribution of the variables was determined using the Kolmogorov–Smirnov test. Considering the independence of the study groups, a parametric *t*‐test was used to compare variables in two groups, and ANOVA was used to compare variables in more than two groups. If the distribution of variables is not normal, the Mann–Whitney non‐parametric test was used to compare variables in two groups, and the Kruskal–Wallis nonparametric test was used to compare variables in more than two groups. Chi‐square test was used for statistical analysis of qualitative variables in groups. *p* value less than 0.05 was considered a significant level.

## 3 Results

Seventy‐two patients were evaluated in this study. A total of 16 patients were excluded due to the lack of inclusion criteria, and 4 patients were excluded due to refusal. After considering the exclusion and inclusion criteria, 52 patients were equally divided into 2 groups in this study, but 1 patient from group C voluntarily underwent surgery due to the lack of expected recovery, and 1 patient from group T did not follow‐up (Figure 1). Demographic information, pain score, ODI, and analgesic consumption are shown in Table 1.

**TABLE 1:** Demographic data, visual analog scale (VAS), Oswestry Disability Index (ODI), and (involved levels, target roots, analgesic consumption, and repeated procedure).

	C group	T group	*p* value
Variable	(Mean ± SD)/*n* (%)	(Mean ± SD)/*n* (%)	
Age (years)	53.9 ± 11.0	51.7 ± 16.7	0.489
Gender			
Male	12 (48%)	11 (44%)	0.657
Female	13 (52%)	14 (56%)	
BMI (kg/m^2^)	28.5 ± 2.4	29.2 ± 2.7	0.589
Involved level			
1	16 (64%)	14 (56%)	0.823
2	9 (36%)	11 (44%)	0.785
Target root			
L3	3 (12%)	3 (12%)	1.000
L4	10 (40%)	9 (36%)	0.560
L5	12 (48%)	14 (56%)	0.257
S1	9 (36%)	10 (40%)	0.560
VAS			
0	7.1 ± 2.2	6.9 ± 2.5	0.442
2nd w.	5.2 ± 2.4	5.5 ± 2.9	0.313
1st m.	5.1 ± 2.4	5.4 ± 2.3	0.591
3rd m.	3.8 ± 2.5	2.8 ± 2.2	0.282
ODI			
0	22.8 ± 11.6	21.8 ± 12.3	0.561
2nd w.	21.1 ± 10.9	18.8 ± 10.6	0.443
1st m.	13.6 ± 10.7	12.7 ± 7.2	0.253
3rd m.	12.9 ± 11.5	11.0 ± 8.6	0.113
Daily analgesic consumption			
Diclofenac (mg)	45 ± 10	42 ± 9	0.442
Acetaminophen (mg)	750 ± 150	700 ± 160	0.226

Pain score and ODI have decreased significantly in both groups, but no difference was observed between the two groups. The frequency of positive Lasègue test was not different in the two groups at the baseline, the second week and the first month, but it was higher in group C than group T in the third month (Table 2). The frequency of other positive tests of Bragard, Psoas, springing, tap, heel walking, tip toe, and stoop did not show any significant difference in the two groups and in any of the time intervals (*p* > 0.05) (Table 2). No significant difference was observed for the interaction effect of time with physical examinations (*p* = 0.42). There was no difference in the amount of analgesic consumption between the two groups. No complications were observed in the two groups, and none of the patients needed to repeat the injection.

**TABLE 2:** Comparison of physical examinations.

Variable	C group (%)	T group (%)	*p* value
Lasègue			
0	18 (72%)	15 (60%)	0.224
2nd w.	4 (16%)	3 (12%)	0.197
1st m.	5 (20%)	3 (12%)	0.086
3rd m.	6 (24%)	4 (16%)	0.043
Bragard			
0	12 (48%)	10 (40%)	0.212
2nd w.	2 (8%)	3 (12%)	0.166
1st m.	3 (12%)	3 (12%)	0.458
3rd m.	5 (28%)	4 (16%)	0.216
Bonnet			
0	5 (20%)	5 (20%)	0.418
2nd w.	1 (4%)	1 (4%)	0.215
1st m.	1 (4%)	0	0.689
3rd m.	0	0	0.986
Faber			
0	7 (28%)	9 (%36)	0.115
2nd w.	1 (4%)	2 (%8)	0.256
1st m.	2 (%8)	2 (%8)	0.355
3rd m.	0	0	1.000
Psoas			
0	12 (48%)	13 (52%)	0.559
2nd w.	2 (8%)	3 (12%)	0.091
1st m.	3 (12%)	2 (8%)	0.058
3rd m.	0	0	1.00
Tap			
0	11 (44%)	8 (32%)	0.112
2nd w.	2 (8%)	2 (8%)	0.096
1st m.	3 (12%)	1 (4%)	0.067
3rd m.	0	0	1.000
Springing			
0	9 (36%)	11 (44%)	0.235
2nd w.	1 (4%)	1 (4%)	0.287
1st m.	1 (4%)	0	0.433
3rd m.	1 (4%)	0	0.433
Tip‐toe walking			
0	6 (24%)	5 (20%)	0.136
2nd w.	2 (8%)	1 (4%)	0.616
1st m.	1 (4%)	0	0.155
3rd m.	0	0	1.000
Heel walking			
0	3 (12%)	4 (16%)	0.537
2nd w.	2 (8%)	2 (8%)	0.267
1st m.	1 (4%)	0	0.112
3rd m.	0	0	1.000
Stoop			
0	4 (16%)	3 (12%)	0.537
2nd w.	0	0	1.000
1st m.	0	0	1.000
3rd m.	0	0	1.000

## 4 Discussion

In this study, the effects of transforaminal and caudal steroid injections using a targeted Racz catheter in the control of lumbar radiculopathy were investigated in a 3‐month period. Pain score (VAS) and Oswestry low back pain disability questionnaire (ODI) were similar. All physical examinations in this period were similar to each other, except for the number of positive Lasègue test in the third month in the caudal group, which was more than the transforaminal. Therefore, it can be raised that the transforaminal approach may be preferable to the caudal approach.

Several studies have compared the effects of different methods of epidural steroid injection in the management of low back pain (Chang‐Chien et al. 2014; Ghai et al. 2014; Makkar et al. 2019; Ozturk et al. 2023). In a comparative study, epidural administration of dexamethasone and hyaluronidase using the Racz technique was compared with transforaminal administration of triamcinolone in controlling pain caused by LDH at lower lumbosacral levels. In this study, pain score (VAS) and functional rate index (FRI) were followed up to 8 weeks (Kim et al. 2013). In their study, VAS and FRI were reduced in both groups, but there was no statistical difference between the two groups. Therefore, they concluded that the transforaminal method is more economical and can be preferred over the caudal neuroplasty. The results of our study were consistent with their study, but the difference in our method was that, unlike their study, the prescribed medication was the same in both groups, and the evaluation of physical examinations was done in a longer follow‐up period (3 months).

In a systematic review and meta‐analysis, the effectiveness of transforaminal versus caudal epidural steroid injection for the management of lumbosacral radicular pain was investigated (Liu et al. 2016). In this review, 6 prospective studies and 2 retrospective studies, including 664 patients, were included. The results of their meta‐analysis showed that, although pain reduction and functional improvements were greater in transforaminal epidural steroid injection than in the caudal method, they were not clinically and statistically significant. Therefore, they concluded that both transforaminal and caudal methods are effective in reducing pain and improving functional levels and have similar effectiveness in managing lumbosacral radicular pain.

The clinical effectiveness of interlaminar and transforaminal epidural steroid injection in LDH was evaluated in another review study (Lee et al. 2018). The results of this study showed that in the transforaminal method, the effectiveness of pain control in the short term and functional improvement in the long term was better than the interlaminar method.

There are studies that have reported similar effects of transforaminal and caudal steroid injections. Three randomized controlled clinical trials have compared three methods of transforaminal, interlaminar, and caudal epidural injection (prescription of local anesthetic with or without steroid) in patients with LDH. The results of these studies showed similar efficacy for caudal, interlaminar, and transforaminal approaches in the management of chronic pain and disability caused by disc herniation (Manchikanti et al. 2015). On the other hand, there are studies that show the effects of transforaminal steroid injection in the management of radicular back pain better than the caudal method. The results of a study conducted in this field showed that in a 3‐month follow‐up, the average VAS and ODI for patients in the transforaminal group were significantly lower compared to the caudal group. From the findings of this study, it was concluded that the transforaminal approach is more effective than the caudal approach (Iqbal et al. 2020).

In another study, the transforaminal method had better results than the caudal method in the management of back pain. In this study, caudal epidural versus transforaminal steroid injection was investigated for short‐term pain relief (6 months) in patients with sciatica caused by lumbar spinal stenosis with single dermotomal distribution in two hospitals. Their results showed that a significant number of spinal stenosis patients had better pain relief 6 months after transforaminal steroid injection (90%) compared to caudal (54%). All patients in the transforaminal group showed functional improvement at 6 months, whereas only three (27%) patients in the caudal group improved functionally. From the findings of this study, it was concluded that the effectiveness of transforaminal steroid injection in patients with sciatica pain caused by spinal stenosis was better than caudal in a 6‐month follow‐up (Ploumis et al. 2014). In our study, caudal steroid injection was performed for one or two levels, with a targeted catheter for getting the medication to the affected area and performing physical examinations. One of the challenges of caudal steroid injection is to reach the affected area exactly, which is possible in the lower lumbosacral levels, but not reliable in the middle and upper lumbar levels. Therefore, a reliable approach to reach the drug in these cases should be considered, because perhaps one of the reasons for the higher efficiency of the transforaminal method is due to the precise administration of the drug in the same area.

Epidural fibrosis is one of the main causes of post‐lumbar surgery syndrome. Steroid injection can be useful in reducing epidural adhesion and fibrosis. A study has compared two methods of transforaminal and caudal steroid injection (triamcinolone 40 mg) in the control of pain syndrome after lumbar surgery. Their results showed that pain scores (NRS‐11) and modified ODI decreased significantly in both groups at 3‐month follow‐up, but the improvement in modified ODI score in Week 3 was better in the transforaminal group (Celenlioglu et al. 2022). The differences between our study and this study included steroid injection at one or two levels, reducing the steroid dose at one level, performing caudal injection with a targeted catheter, and performing physical examinations. In some studies, there is still the challenge of caudal steroid injection without the use of a targeted catheter and its reduced efficacy compared to transforaminal injection. In addition, the lack of physical examination can be one of the weak points of some studies. Although the pain score and disability index were used as common criteria in evaluating patients in similar studies, one of the strengths of our study was performing several physical examinations in patients, which was not done in many studies. The pain score and the disability scale must be expressed by the patient and are subjective tools, so many intervening factors such as the patient's culture and pain threshold can affect it (Tschugg et al. 2016).

Some studies have shown that neurological tests have high sensitivity and specificity in diagnosing LDH (Majlesi et al. 2008). After the definitive diagnosis of LDH and lumbar radiculopathy, performing neurological tests and physical examinations may have a special position in the treatment process along with other modalities (such as pain score and ODI) (Samolsky Dekel et al. 2020). Conducting physical examinations as an objective tool may be useful in reducing the error rate of subjective tools, such as VAS and ODI, and help in increasing the accuracy of study results. Choosing the best physical examination or a set of examinations in these patients needs more studies. The limitation of this study was examining patients in only one medical center with a small sample size and a short follow‐up period.

## 5 Conclusion

The findings of this study showed that during a 3‐month follow‐up, transforaminal and caudal epidural injections using a targeted catheter have the same effectiveness in controlling pain and improving the functional disability of patients. Since during the follow‐up period with physical examinations, only the positive Lasègue test in the caudal group was more than the transforaminal method in the long term, it may be possible that the transforaminal method is preferable to the caudal one.

## Author Contributions

**Farnad Imani**: conceptualization, investigation, project administration, supervision; writing—review and editing. **Faezeh Mohammad‐Esmaeel**: data curation, investigation, writing—original draft. **Seyedeh‐Fatemeh Morsalli**: data curation, investigation, methodology. **Ali Ahani‐Azari**: investigation, methodology, validation. **Mahzad Alimian**: data curation, investigation, methodology. **Nasim Nikoubakht**: Formal analysis, investigation, writing—original draft. **Azadeh Emami**: formal analysis, investigation, visualization.

## Ethics Statement

The study was approved by the Ethics Committee of the Iran University of Medical Sciences (Code: IR.IUMS.FMD.REC.1399.563; Approval date: 2021‐01‐11, 1399/10/22).

## Consent

Informed written consent was obtained from all patients.

## Conflicts of Interest

The authors declare no conflicts of interest.

### Peer Review

The peer review history for this article is available at https://publons.com/publon/10.1002/brb3.70067.

## Data Availability

The dataset presented in the study is available upon request from the corresponding author during submission or after publication. The data are not publicly available due to privacy reasons, and further investigations are in progress on these data.
